# Conversion of Broad-Spectrum Antimicrobial Peptides into Species-Specific Antimicrobials Capable of Precisely Targeting Pathogenic Bacteria

**DOI:** 10.1038/s41598-020-58014-6

**Published:** 2020-01-22

**Authors:** Lin Xu, Changxuan Shao, Guoyu Li, Anshan Shan, Shuli Chou, Jiajun Wang, Qingquan Ma, Na Dong

**Affiliations:** 1 Institute of Animal Nutrition, Northeast Agricultural University, 600 Changjiang Road, Harbin, China; 2 Heilongjiang Polytechnic, 5 Xuefu Road, Harbin, China

**Keywords:** Peptides, Antibiotics

## Abstract

Currently, the majority of antibiotics in clinical use have broad activity spectra, killing pathogenic and beneficial microorganisms indiscriminately. The disruption of the ecological balance of normal flora often results in secondary infections or other antibiotic-associated complications. Therefore, targeted antimicrobial therapies capable of specifically eliminating pathogenic bacteria while retaining the protective benefits of a normal microflora would be advantageous. In this study, we successfully constructed a series of *Enterococcus faecalis*-targeted antimicrobial peptides from wide-spectrum antimicrobial peptide precursors. These peptides are designed based on fusion of the species-specific peptide pheromone cCF10 and modification of the active region of the antimicrobial peptide. The results showed that cCF10-C4 possessed specific antimicrobial activity against *E*. *faecalis* and was not active against other types of bacteria tested. The specificity of this hybrid peptide was shown by the absence of antimicrobial effects in the pheromone-substituted derivative. Further studies indicated that cCF10-C4 and its parent peptide C4 exert their activities by damaging cytoplasmic membrane integrity. The present study reveals the application potential of these molecules as “probiotic” antimicrobials for the control of specific bacterial infections, and it also helps to elucidate the design and construction of species-specific antimicrobials with precise targeting specificity.

## Introduction

The mucosal surfaces and skins of animals are colonized by plenty of microorganisms. Most of the bacteria within the multispecies microbial community are beneficial^1^. This indigenous flora plays a very important role in nutrient acquisition and protective colonization^2–4^. Additionally, the normal flora represents an important ecological system, and alterations to the microflora may result in bacterial infections^5–7^. Unfortunately, the majority of conventional antibiotics have wide activity spectra, killing pathogens and normal microflora indiscriminately and disrupting the micro-ecological balance. The unavoidable loss of microflora and ecological disruption resulting from antibiotic treatment may lead to severe and recurrent complications from persistent pathogens or opportunistic microorganisms that recolonize the vacated niche easily^8^. The imbalance of normal microflora and the increasing threat of multidrug-resistant microbes highlight the urgent need for novel “targeted” antimicrobial therapies that can selectively eliminate pathogens without significantly disrupting resident normal flora.

Antimicrobial peptides (AMPs) found in host immune systems have attracted considerable attention due to their excellent antimicrobial properties and unique action mechanism. These peptides are broad-spectrum with potent activities against microbes, viruses, parasites and even tumor cells^9–11^. Furthermore, unlike traditional antimicrobial agents that inhibit specific metabolic pathways, the majority of antimicrobial peptides exert bactericidal effects via the irreversible disruption of bacterial membranes, inducing the leakage of vital cell components and resulting in eventual cell death^12,13^. The physical nature of membrane disruption is expected to prevent or delay the development of bacterial resistance to AMPs^14^. Due to these inherent advantages, AMPs can be used as effective weapons against pathogenic microorganisms such as multidrug-resistant microbes and are amongst the most promising candidates for a new generation of antimicrobial agents.

The idea of targeted antimicrobial therapy is supported by several naturally occurring AMPs, including plantaricin, lantibiotic and nisin. These AMPs were found to contain both targeting and antimicrobial domains within their peptide sequences^15–17^. The presence of targeting domains promoted the accumulation of peptides on the bacterial membrane, increasing local peptide concentrations and enhancing bactericidal activities against the targeted microbes^18,19^. In recent years, several attempts have been made to construct pathogen-selective antimicrobials by conjugating a bacterial recognition domain to antimicrobial peptides or bactericidal proteins^20–25^. These findings provided an intriguing starting point for the development of multidomain AMPs with selective bactericidal effects against specific bacteria. Although all of these synthetic molecules exhibited enhanced antimicrobial potency, selectivity, and kinetics against specific bacteria, inevitable bactericidal effects on unrelated bacteria were also observed with these peptides. The main reason for this problem may lay in the fact that the killing moiety of these fusion peptides had not been redesigned or modified, and thus electrostatic attractions between cationic peptide molecules and anionic lipids of the bacterial membrane were retained, thereby causing indiscriminant bactericidal effect via a non-receptor-mediated pathway. Thus, these approaches have yet to generate target-specific antimicrobials.

In this study, a rational approach was adopted to construct a series of *Enterococcus faecalis*-targeted AMPs using the enterococcal pheromone cCF10 as the targeting domain and optimizing the active centre of AMP for enhanced target specificity. Bacterial peptide pheromones are species-specific signalling molecules that mediate intercellular communication in some Gram-positive bacteria and fungi^26^. These small peptides can traverse the cell wall and bind to the cognate membrane receptors with high affinities^27,28^. The targeting specificity and remarkable affinity of pheromones to their membrane receptors make them ideal candidates for species-specific targeting peptide domains that provide specific binding to a selected organism. The antimicrobial activities and targeting specificities of the designed peptides were evaluated against a panel of microbial species including both Gram-negative and Gram-positive bacteria. The haemolytic properties these peptides were measured, and flow cytometric, fluorescent spectrographic, and electron microscopy assays were performed to further evaluate peptide-membrane interaction mechanisms. The aim of the present study is to provide a rational approach to convert normally wide-spectrum antimicrobial peptides into “smart” targeted antimicrobials with precise specificity against target organisms without damaging benign bystanders.

## Results

### Peptide design and characterization

Initially, targeted antimicrobial peptide molecules were constructed by fusing the *E*. *faecalis*-specific peptide pheromone cCF10 with the broad-spectrum antimicrobial peptide C6, derived from the designed tryptophan-zipper antimicrobial peptide WK3 at either the N-terminus or the C-terminus^29^. To ensure that the targeting domain and the killing domain remain functionally and structurally independent, a flexible peptide linker (GGG) was incorporated between the two functional domains. Biological testing of these peptides showed that the addition of cCF10 at the N terminus of C6 significantly increased antimicrobial activity against *E*. *faecalis* relative to C6 alone; however, the hybrid peptides still possessed antimicrobial activities against the other bacteria tested. We reasoned that these undesired bactericidal effects against unrelated bacteria might arise from a high density of positive charges, which contributes to AMPs attraction toward and attachment to anionic bacteria cell membranes. In nature, such interactions occur via nonspecific electrostatic interactions. Given the known high affinity and specificity of a pheromone to its membrane receptor, we theorized that the cCF10 pheromone is adequate for targeted delivery of the antibacterial peptide to *E*. *faecalis*. Weakening the electrostatic interactions between AMPs and microbes may therefore prevent the molecules from entering the microbial membrane, decreasing or abolishing antimicrobial activity against untargeted bacterial species, while maintaining potent antimicrobial activity toward pheromone-sensing bacteria. To test this hypothesis, peptides with decreased positive charges were designed by replacing cationic K residues with anionic E or neutral Q residues at defined positions. The net charge of the peptide was decreased from +6 (cCF10-C6) to +4 (cCF10-C4) and +3 (cCF10-C3). Additionally, to evaluate the pheromone’s role in the specificity of the targeted antimicrobial peptide, the cCF10 targeting domain in cCF10-C4 was replaced with a random peptide sequence of the same length as cCF10.

The key physicochemical parameters of the designed peptides are summarized in Table 1. MALDI-TOF MS analysis showed that the measured molecular weights of the synthetic peptides were in close agreement with their theoretical values, suggesting that the products correspond to the designed compositions.

**Table 1:** Amino acid sequence, molecular weight, and net charge of the designed peptides.

Peptides	Sequence	Molecular mass (Da)		Net charge
		Calculated	Observed	
cCF10	LVTLVFV	790.01	790.02	0
C6	WKWKWKNGKWKWKW	2075.49	2075.52	+6
cCF10-C6	LVTLVFVGGGWKWKWKNGKWKWKW	3018.65	3018.60	+6
C6-cCF10	WKWKWKNGKWKWKWGGGLVTLVFV	3018.65	3018.68	+6
C4	WKWKWENGKWKWKW	2076.44	2076.46	+4
cCF10-C4	LVTLVFVGGGWKWKWENGKWKWKW	3019.59	3019.62	+4
C3	WKWKWENGKWKWQW	2076.39	2076.41	+3
cCF10-C3	LVTLVFVGGGWKWKWENGKWKWQW	3019.55	30.9.58	+3
random-C4	YSTCFIMGGGWKWKWENGKWKWKW	3093.62	3093.66	+4

### Antimicrobial activity

To evaluate the antibacterial property and general specificity of the designed peptides, MIC tests were performed against a broad selection of model bacteria, including Gram-negative bacteria and Gram-positive bacteria. As shown in Table 2, cCF10 had no effect on the growth of the bacterial species tested. C6 exhibited broad-spectrum antimicrobial activity against the panel of microbes, with MICs ranging from 2 to 16 µM. With the fusion of cCF10, the C6-cCF10 peptide showed similar antimicrobial activity against *E*. *faecalis* compared to the parent antimicrobial peptide C6, while cCF10-C6 displayed four-fold increased antimicrobial activity relative to that of C6 alone. However, the abilities of these two hybrid peptides to inhibit the growth other bacteria were much weaker than that of C6 alone. These findings showed the first indications that the presence of the cCF10 targeting domain increased antimicrobial activity and selectivity against *E*. *faecalis* but no other microorganisms. More importantly, the reduction of net positive charges within the killing domain resulted in the loss of bactericidal effects toward all the untargeted bacteria while maintaining robust antimicrobial activity against *E*. *faecalis*: cCF10-C4 exhibited an MIC against *E*. *faecalis* eight times lower than C4 alone, indicating a unique specificity for the targeted bacteria. However, it should be noted that further decreasing the positive charge to +3 abolished antimicrobial activity completely. Furthermore, the substitution of the cCF10 targeting domain with a random peptide sequence led to the loss of antimicrobial activity to *E*. *faecalis* (Table 2). These results indicate that the specificity of cCF10-C4 against the targeted bacteria is dependent on pheromone-receptor interactions.

**Table 2:** MIC of the peptides against gram-negative and gram-positive bacterial strains.

Peptides	MIC (μM) Gram-positive					
	Gram-positive			Gram-negative		
	*E*. *faecalis 25922*	*S*. *aureus 1005*	*S*. *epidermidis 7913*	*S*. *typhimurium 29213*	*S*. *Pullorum 43300*	*E*. *coli 12228*
cCF10	>128	>128	>128	>128	>128	>128
C6	16	4	8	8	4	2
C6-cCF10	16	8	32	>128	8	2
cCF10-C6	4	32	32	>128	16	4
C4	64	32	32	32	8	2
cCF10-C4	8	>128	>128	>128	>128	64
C3	>128	>128	>128	64	16	32
cCF10-C3	>128	>128	>128	>128	>128	>128
random-C4	>128	>128	>128	>128	>128	32
PG-1	4	8	16	8	8	2

### Hemolytic activity

**Figure 1:**
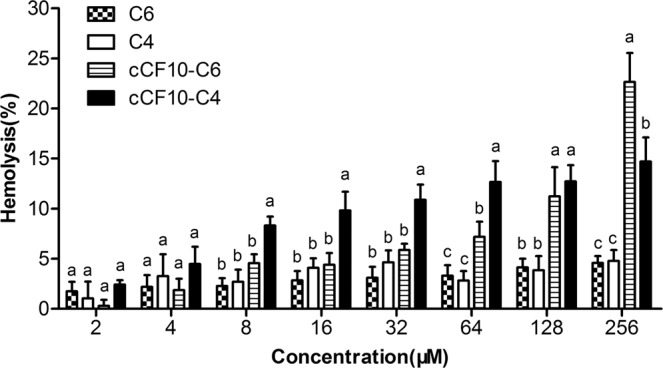
Haemolytic effect of the peptides against hRBCs. Results were presented as the mean ± standard deviation (SD) of three independent assays. Differences between groups exposed to the same peptide concentration were analyzed by one-way ANOVA followed by Turkey’s post-hoc analysis (n = 3). The values with different superscripts (a, b, c) indicate a significant difference (*P* < 0.05).

The haemolytic activities of the fusion peptides against human erythrocytes were evaluated to provide an indication of their cytotoxicity to mammalian cells (Fig. 1). The parent peptides C4 and C6 induced no haemolysis in human red blood cells at all concentrations tested. In contrast, the hybrid peptides cCF10-C4 and cCF10-C6 demonstrated little detectable haemolysis, with MHCs (the concentration that induces 10% or more haemolysis) of 32 µM and 128 µM, respectively. These MHCs indicate that the haemolytic activity of the peptides increases when the cCF10 pheromone is fused to the peptide.

### Cytoplasmic membrane electrical potential

**Figure 2:**
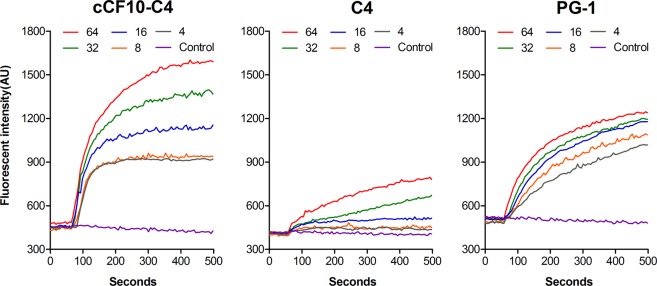
Cytoplasmic membrane depolarization of *E*. *faecalis* ATCC 29212. The cytoplasmic membrane potential variation was evaluated using the membrane potential-sensitive dye diSC3–5. Dye release was monitored at an excitation wavelength of 622 nm and an emission wavelength of 670 nm as a function of time.

Due to the peptide’s greatly enhanced antimicrobial and unique specificity against *E*. *faecalis*, the mechanism of action of the targeted peptide cCF10-C4 was studied. The ability of the peptide to depolarize bacterial cytoplasmic membranes was determined using the membrane potential-dependent probe diSC_3_-5, which is quenched in the cytoplasmic membrane. The cytoplasmic membrane potential dissipates when the membrane is disrupted and permeabilized, releasing diSC_3_-5 into the medium and resulting in a conspicuous increase in fluorescence. The results showed that, the depolarization of the cell membrane of *E*. *faecalis* by the peptides was time- and dose-dependent (Fig. 2). The peptide cCF10-C4 caused a rapid increase in fluorescence at low micromolar concentrations. This increase was obviously higher than in parallel samples treated with the same concentration of C4 parent peptide, indicating that the addition of pheromone to C4 selectively enhanced the capacity to dissipate the transmembrane potential of the target bacteria.

### Flow cytometry

**Figure 3:**
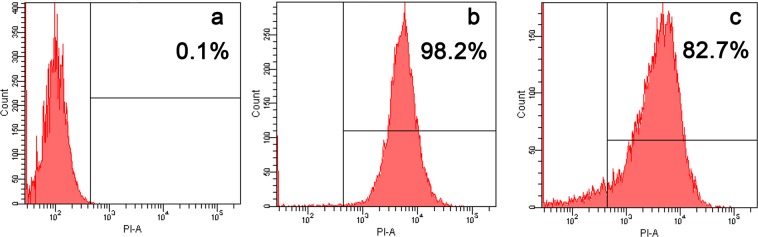
Flow cytometric analysis. The membrane damage of *E*. *faecalis* ATCC 29212 treated by peptides was measured by an increase in the fluorescence intensity of propidium iodide (PI) at 4 °C for 30 min. (**a**) control; (**b**) cCF10-C4 treated; (**c**) C4 treated. Bacteria were treated with peptides at 1 × MICs for 30 min. The control was processed without peptides. Data are representative of three independent experiments.

The DNA-intercalating dye PI was used to evaluate the integrity of the microbial cell membranes. PI stains the nucleic acids in cells when their membranes are disrupted, causing an increase in fluorescence. As shown in Fig. 3, only 0.1% of *E*. *faecalis* cells were stained with PI in the absence of peptide. After treatment with cCF10-C4 and C4 at their minimum inhibitory concentrations, 98.2% and 82.7% of cells presented PI fluorescent signals, respectively. It is clear from the data that both peptides possessed the capability to destruct the cell membrane integrity of *E*. *faecalis*. However, cCF10-C4 caused a greater accumulation of PI than its parent peptide C4.

### Scanning electron microscopy (SEM) observations

**Figure 4:**
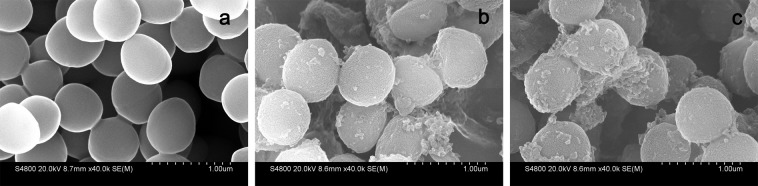
SEM micrographs of *E*. *faecalis*. (**a**) control; (**b**) cCF10-C4 treated; (**c**) C4 treated. *E*. *faecalis* was treated with peptides at 1 × MICs for 120 min. The control was processed without peptides.

SEM was employed to directly visualize bacterial surface morphology and membrane damage after treatment with peptides at 1 × MICs for 2 h. In comparison to the untreated cells, which exhibited smooth and intact surfaces (Fig. 4A), treatment with cCF10-C4 and C4 induced significant morphological alterations. The membrane surfaces of peptide-exposed *E*. *faecalis* cells became roughened, deformed, and covered by numerous blebs (Fig. 4B,C).

### Transmission electron microscopy (TEM) observations

**Figure 5:**
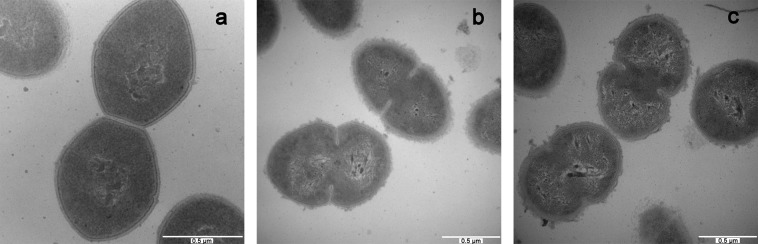
TEM micrographs of *E*. *faecalis*. (**A**) control; (**B**) cCF10-C4 treated; (**C**) C4 treated. *E*. *faecalis* was treated with peptides at 1 × MICs for 120 min. The control was processed without peptides.

To investigate the membrane integrity and intracellular alterations of *E*. *faecalis* cells treated with peptides, samples were analysed by TEM. As shown in Fig. 5, intact membrane envelopes and dense internal structures were observed in the untreated bacterial cells (Fig. 5A). Following treatment with peptides, the cytoplasmic membranes became blurred and began to collapse (Fig. 5B,C). In addition, dispersion of intracellular contents was also observed in peptide-treated *E*. *faecalis* cells.

## Discussion

The overwhelming majority of antimicrobials in clinical use possess wide-spectrum bactericidal activities. These antibiotics have an advantage when the specific pathogen causing an infection is unknown. However, the use of such antimicrobials may indiscriminately kill benign and and pathogenic microbes, inducing an imbalance in the nomal flora and resulting in severe posttreatment complications^17^. Therefore, pathogen-specific antimicrobial therapies capable of precisely killing pathogens without affecting normal flora would be an ideal solution and could help to re-establish ecological balance and provide long-term protection. Unlike conventional antimicrobial agents, which may lose effect when their molecular structures are changed, AMPs can be optimized to obtain desirable properties through modification of their primary sequences^30,31^, making them a potential treasure trove of starting points for the development of targeted antimicrobial biomaterials. Here, we successfully designed a targeted antimicrobial peptide with unique specificity against a target organism without damaging other types of bacteria. The peptide was constructed by incorporating a natural pheromone as the targeting domain and reducing the net positive charge within the killing domain for enhanced target specificity. Given the universality and scalability of this approach, we believe that the methods presented here would be an effective strategy to make target-specific therapies a reality.

Our results showed that the fusion of the targeting region to the N-terminus of the antimicrobial peptides provided a clear increase in antimicrobial activity against the targeted bacteria. Given the high affinity of pheromones for their membrane receptors, it is likely that the improved activities of the hybrid peptides arise from selective accumulation of the peptide molecules on *E*. *faecalis* membranes, mediated by the pheromone cCF10. This accumulation generates higher local peptide concentrations, thereby enhancing antibacterial activity^23^. In addition, it is possible that the hydrophobicity of the fused cCF10 (containing 6 hydrophobic residues) may enhance the intrinsic ability of the molecules to insert into the cytoplasmic membrane bilayer independent of receptor binding. However, we found that all the fusion peptides exhibited decreased antimicrobial activities compared to their parent peptides against the other untargeted microorganisms tested. These findings suggest that the pheromone-receptor interaction is the primary reason for the increased antimicrobial activities. Further studies demonstrated that substitution of cCF10 with a random sequence resulted in a complete loss of bactericidal effects. The results further validate the notion that the specific antimicrobial activity of cCF10-C4 against the targeted bacteria is receptor-dependent. Both cCF10 and C4 components were necessary; however, neither component alone was sufficient. It has been reported that the cCF10 pheromone binds to the PrgZ receptor at the N-terminus^28^. As observed in this study, cCF10-C6 with a free pheromone N-terminus exhibited robust antimicrobial activity against *E*. *faecalis*; the activity of C6-cCF10 was much weaker in comparison, indicating that the pheromone-receptor interaction may be affected by where C6 was fused.

It is generally agreed that the first step in AMP activity is binding of cationic residues in the peptides to the anionic lipids of the microbial membrane via electrostatic interactions^32^. Previous literature has demonstrated that within a threshold (usually +6), increasing the positive charge of an antibacterial peptide facilitates its anchoring to the membrane, resulting in improved antibacterial properties^33,34^. Both of the initially designed peptides (cCF10-C6 and C6-cCF10) contained high net charges (+6), indicating sufficient driving of the electrostatic attachment to the bacterial membrane. Thus it was not surprising to find that these fused peptides still retained antimicrobial activities against unrelated bacterial strains. As a targeting domain, the pheromone cCF10 provides specific binding to its target organism with high affinity independent of electrostatic attraction. Therefore, to further narrow the antibacterial spectrum of the peptide, we decreased the net positive charge within the killing domain. Our data showed that the peptide cCF10-C4 is capable of precisely killing *E*. *faecalis* without affecting any of the other microorganisms tested. Therefore, we believe that our goal to construct a specifically targeted antimicrobial peptide was achieved. Furthermore, the specificity of targeted antibacterial peptides can be increased by reducing positive charges within a certain range. Further decreasing the net charge to +3 (cCF10-C3) resulted in the loss of antimicrobial effects, implying that the antimicrobial peptide domain was not sufficient to disrupt bacterial membranes. This finding provides evidence that the cationic amino acid residues within AMPs play important roles not only in the electrostatic interactions of peptides with anionic lipids but also in the physical disruption of microbial cell membranes. Further studies are underway to elucidate the implications of this new finding.

Hemolysis is often thought to be one of the obstacles preventing the clinical applications of AMPs as antibacterial agents. Previous literatures showed that peptide hydrophobicity is positively correlated with haemolytic activity^29,35^. Consistent with this interpretation, the addition of the cCF10 pheromone to C6 or C4 caused a slight increase in haemolytic rates. Fortunately, the hybrid peptides induced minimal or no haemolysis against human red blood cells at antimicrobial levels, indicating that these peptides could be developed as potential antibacterial agents for clinical use.

According to previous studies, the majority of cationic AMPs exert antimicrobial activities by damaging cytoplasmic membrane integrity^13,36^. Therefore, in this work, the bactericidal mechanism was evaluated, placing a special focus on the influences of peptides on the cytoplasmic membrane. Generally, membrane depolarization is an obvious feature of peptide-membrane interactions that plays an important role in determining the antibacterial potency of AMPs^37^. Our results indicated that the cCF10-C4 peptide results in a significant increase in the degree of cytoplasmic membrane depolarization in *E*. *faecalis* compared to C4 alone (Fig. 2), which correlates with their antibacterial activities against the targeted bacteria. This property was not observed in tests of untargeted bacterial species, implying that the enhanced killing activity and target specificity of the hybrid peptide may result from increased depolarization of the bacterial membrane. Furthermore, the results from TEM and SEM studies demonstrated that the designed peptides exert their bactericidal activities by damaging cytoplasmic membranes, causing the leakage of cytoplasmic components into the extracellular medium (Fig. 4 and Fig. 5). In addition, flow cytometry data further demonstrated that the designed peptides killed bacteria by disrupting cell membrane integrity (Fig. 3). This mechanism of action, based on physical destruction of the membrane, make it difficult for microbes to acquire resistance because this would require bacteria to mutate or repair their entire membrane lipid composition.

## Conclusions

In this work, a series of targeted antimicrobial peptides were constructed and evaluated for their biological activities and antimicrobial mechanisms. The peptide was designed by fusing a natural pheromone as the targeting domain and reducing positive charge for enhanced target specificity. The designed peptides, in particular cCF10-C4, exhibited robust specific activity against targeted *E*. *faecalis* at micromolar concentrations, while pheromone-insensitive organisms were not affected. This specificity, combined with the low hemolytic activity of the designed peptides, suggests that these peptides can be used as promising antimicrobial agents for clinical applications against specific bacterial infections. Additionally, cCF10-C4 exerted its bactericidal effects via the destruction of cytoplasmic membrane integrity, allowing the efflux of internal components and resulting in cell death. This physical membrane disruption mechanism may decrease the occurrence of microbial resistance. Taken together, our findings presented here provides a feasible strategy for the construction of targeted AMPs with precise specificity against target organism without damaging other bacterial species. Furthermore, peptide pheromones widely existed in microorganisms, implying a large pool which provide growing candidates for the development of novel targeting peptides. This indicates that targeted antimicrobial agent construction in the future will be a “tunable” process whereby a wide variety of combinations of targeting and killing regions may be combined.

## Materials and Methods

### Materials

The bacterial strains *Enterococcus faecalis* ATCC 29212, *Staphylococcus epidermidis* ATCC 12228, *Staphylococcus aureus* ATCC 29213, *Salmonella typhimurium* C7731, *Escherichia coli* ATCC 25922 and *Salmonella Pullorum* C7913 were obtained from the College of Veterinary Medicine, Northeast Agricultural University (Harbin, China). The human red blood cells (hRBCs) were obtained from the Northeast Agricultural University Hospital.

Phosphate-buffered saline (PBS) solution was purchased from Kermel (China). Mueller Hinton Broth (MHB) powder was purchased from AoBoX (China) and used to prepare the microbial broths according to the manufacturer’s instructions. 3,3′-dipropylthiadicarbocyanine (diSC_3_-5), dimethyl sulfoxide (DMSO), ethanol (analytical grade, 99%), tertiary butanol (analytical grade, 99%), acetone (analytical grade, 99%), glutaraldehyde (synthetic grade, 50% in H_2_O) and propidium iodide (PI) were all ordered from Sigma-Aldrich (China).

### Peptides synthesis

The peptides used in this study were synthesized and purified by GL Biochem (Shanghai, China) through solid-phase methods using Fmoc chemistry. Matrix-assisted laser desorption/ionization time-of-flight mass spectroscopy (MALDI-TOF MS, Linear Scientific Inc., U.S.A.) was used to determine the true molecular masses of these peptides. Peptide purities were confirmed to be >95% using analytical reverse-phase high-performance liquid chromatography (HPLC). The peptide cCF10 was dissolved in 40% DMSO, and other peptides were dissolved in deionized water at a concentration of 2.56 mM. Peptides were stored at −20 °C for subsequent assessments.

### Antimicrobial assays

The antimicrobial activities of the peptides were investigated against the following bacteria: *E*. *faecalis* ATCC 29212, *S*. *epidermidis* ATCC 12228, *S*. *aureus* ATCC 29213, *E*. *coli* ATCC 25922, *S*. *typhimurium* C7731 and *S*. *Pullorum* C7913. The MICs of the peptides were determined using a modified standard microtiter dilution method as described previously^29,38^. Briefly, the microbial strains were cultured in MHB at 37 °C to reach mid-log phase and then diluted to 0.5-1×10^6^ CFU/ml. 50 μL volumes of bacteria suspension were incubated in sterile 96-well plates with 50 μl of 2-fold serially diluted peptides at different concentrations (0.25–128 μM). Cultures with or without bacterial cells was employed as positive and negative controls, respectively. MIC was defined as the minimal concentration of peptides that results in no visible turbidity after incubation at 37 °C for 16 h. Each measurement was reproduced at least four times using three replicates.

### Hemolytic activity assay

The hemolytic activity of the peptides was evaluated as the amount of hemoglobin released by the disruption of human red blood cells (hRBCs)^39,40^. Fresh human erythrocytes were obtained from a healthy donor (Changxuan Shao, Harbin, China) after informed consent. Then, the collected hRBCs were washed three times with PBS (pH 7.2), centrifuged at 1,000 × g for 5 min at 4 °C, and re-suspended in PBS to attain a dilution of approximately 1% (v/v) of erythrocytes. A 50 μl portion of the erythrocyte suspension were incubated with 50 μl of serially diluted peptides (0.5–256 μM) dissolved in PBS for 1 h at 37 °C. After centrifugation (1,000 × g, 5 min, 4 °C), the supernatant was transferred to a new 96-well plate. Haemoglobin release was measured by monitoring optical density (OD) at 570 nm. The hRBCs in PBS and 0.1% (v/v) Triton X-100 were employed as negative and positive controls, respectively. Minimal haemolytic concentrations (MHCs) were defined as the peptide concentrations causing 10% haemolysis. Three independent experiments were performed in duplicate. The percent hemolysis was calculated using the following equation: Hemolysis (%) = [(OD570 of the treated sample − OD570 of the negative control)/(OD570 of the positive control − OD570 of the negative control)] × 100%. The experimental protocol was reviewed and approved by the ethics committee of the Northeast Agricultural University Hospital, and the experimental method was carried out in accordance with the approved guidelines and regulations.

### Cytoplasmic membrane depolarization assay

The membrane depolarization activity of the peptides was evaluated using the membrane potential-sensitive fluorescent dye diSC_3_-5 as described previously^36^. Briefly, *E*. *faecalis* ATCC 29212 cells were grown to mid-log phase, harvested by centrifugation at 1,000 × g for 10 min, washed thrice, and re-suspended to an OD_600_ of 0.05 with 5 mM HEPES buffer (pH 7.4, containing 20 mM glucose) containing 0.1 M KCl to equilibrate cytoplasmic and external K^+^ concentrations. The cell suspensions were then incubated with 0.4 μM diSC_3_-5 for 90 min. Subsequently, 2 mL of cell suspension was added to a 1 cm quartz cuvette and mixed with peptides at final concentrations ranging from 4 to 64 µM. Changes in fluorescence were recorded (excitation λ = 622 nm, emission λ = 670 nm) with an F-4500 Fluorescence Spectrophotometer (Hitachi, Japan). The deionized water without peptides and natural βhairpin AMP PG-1 were employed as negative and positive controls, respectively.

### FACScan analysis

Bacterial cell membrane integrity was evaluated by flow cytometry^38^. Briefly, *E*. *faecalis* ATCC 29212 was grown to mid-log phase, harvested, washed thrice with 10 mM PBS and diluted to 10^5^ CFU/ml. Bacterial suspensions were incubated with peptides at their minimum inhibitory concentrations for 30 min at 28 °C. Propidium iodide (PI, Sigma) was added at a final concentration of 10 µg/ml and incubated for an additional 30 min at 4 °C. Unbound dye was removed by washing with excess PBS. Cells incubated with PI in the absence of peptides were served as the negative control. Data were recorded using a FACScan instrument (Becton-Dickinson, San Jose, CA) with a laser excitation wavelength of 488 nm.

### Scanning electron microscopy (SEM) characterization

Bacteria samples were prepared as described previously^38^. Briefly, *E*. *faecalis* ATCC 29212 cells were cultured in MHB at 37 °C to mid-log phase, harvested by centrifugation at 1,000 × g for 10 min, washed twice with 10 mM PBS, and re-suspended to an OD_600_ of 0.2. The cell suspensions were incubated at 37 °C for 120 min with different peptides at their 1× MICs. Controls were run without peptides. Following the incubation, the cells were centrifuged and washed thrice with PBS at 5,000 × g for 5 min. Subsequently, the bacterial cell pellets were fixed overnight with 2.5% (v/v) glutaraldehyde at 4 °C and washed twice with PBS followed by dehydration in a graded ethanol series (50%, 70%, 90%, and 100%) for 15 min each. The dried specimens were transferred to a mixture of ethanol and tertiary butanol (1:1, v/v) for 20 min followed by pure tertiary butanol for 30 min. Finally, the samples were dried using a critical point dryer, coated with gold, and then observed by SEM (Hitachi S-4800, Japan).

### Transmission electron microscopy (TEM) characterization

TEM was performed to visualize intracellular alterations as described previously^40,41^. Bacteria samples were initially prepared as for SEM. After pre-fixation with 2.5% glutaraldehyde overnight, the cell pellets were washed twice and were post-fixed with 2% osmium tetroxide for 90 min. After being washed twice with PBS, the samples were dehydrated for 10 min in a graded ethanol series (50%, 70%, 90%, and 100%) followed by 10 min in a mixture of absolute ethanol and acetone (1:1, v/v) and 10 min in absolute acetone. Subsequently, the specimens were transferred to a mixture of absolute acetone and epoxy resin (1:1, v/v) for 30 min and then to pure epoxy resin overnight at a constant temperature. Finally, the samples were sectioned using an ultramicrotome, stained with uranyl acetate and lead citrate, and then observed by TEM (Hitachi H-7650, Japan).

### Statistical analysis

Data were analysed by one-way ANOVA using SPSS 16.0 software. Quantitative data are presented as the means ± standard deviation. Differences were defined as significant at a *P*-value of less than 0.05.

## Data Availability

The datasets used and/or analysed during the current study are available from the corresponding author on reasonable request.
